# A Survey on Underwater Wireless Sensor Networks: Requirements, Taxonomy, Recent Advances, and Open Research Challenges

**DOI:** 10.3390/s20185393

**Published:** 2020-09-21

**Authors:** Salmah Fattah, Abdullah Gani, Ismail Ahmedy, Mohd Yamani Idna Idris, Ibrahim Abaker Targio Hashem

**Affiliations:** 1 Department of Computer System and Technology, Faculty of Computer Science and Information Technology, University of Malaya (UM), Kuala Lumpur 50603, Malaysia or salmahf@ums.edu.my (S.F.); ismailahmedy@um.edu.my (I.A.); yamani@um.edu.my (M.Y.I.I.); 2 Faculty of Computing and Informatics, Universiti Malaysia Sabah (UMS), Kota Kinabalu 88400, Sabah, Malaysia; 3 College of Computing and Informatics, Department of Computer Science, University of Sharjah, Sharjah 27272, UAE; ihashem@sharjah.ac.ae

**Keywords:** underwater sensor networks, acoustic communication, ocean environment, wireless sensor networks, UWSN

## Abstract

The domain of underwater wireless sensor networks (UWSNs) had received a lot of attention recently due to its significant advanced capabilities in the ocean surveillance, marine monitoring and application deployment for detecting underwater targets. However, the literature have not compiled the state-of-the-art along its direction to discover the recent advancements which were fuelled by the underwater sensor technologies. Hence, this paper offers the newest analysis on the available evidences by reviewing studies in the past five years on various aspects that support network activities and applications in UWSN environments. This work was motivated by the need for robust and flexible solutions that can satisfy the requirements for the rapid development of the underwater wireless sensor networks. This paper identifies the key requirements for achieving essential services as well as common platforms for UWSN. It also contributes a taxonomy of the critical elements in UWSNs by devising a classification on architectural elements, communications, routing protocol and standards, security, and applications of UWSNs. Finally, the major challenges that remain open are presented as a guide for future research directions.

## 1. Introduction

Wireless sensor networks (WSN) have considerable potential in monitoring aquatic environments by sensing, collecting, and transferring data wirelessly to users in real time. It has indirectly led to the emergence of a new paradigm of wireless sensor technology known as underwater wireless sensor networks (UWSNs). UWSN technologies are implemented and deployed in deep underwater with sensors using acoustic signals to perform communication.

Although UWSNs have exhibited a significant growth in the world market, a diverse range of application requirements have contributed to the pressing constraints on the performance of network tasks, and the capability of sensor nodes deployed in the monitored region. Furthermore, the available UWSN sensor nodes in the market have limited power and capacity, channel reliability, and complexity in acoustic signal communications [1,2]. Therefore, the underwater wireless sensor network theories and applications are still required further exploration and review.

Table 1 summarizes the existing recent relevant studies and highlights the research gap. However, these surveys covered only some basic features that still need to be updated based on the current research, technological advances, and new applications. From Table 1, it is concludable that many studies have failed to consider the crucial aspects in UWSNs. Hence, this study is conducted with the aim of investigating the domain of underwater wireless sensor networks and provide comprehensive insight into UWSNs requirements, platforms, recent advances, taxonomy and challenges of UWSNs. Additionally, this paper offers the newest evidence for various aspects that can satisfy the requirements for rapid development of UWSNs.

The contributions of this paper are summarized as follows:

- Present a brief compilation of motivation for UWSNs and its significance.
- Identify and describe the key requirements to achieve essential procedures of implementing UWSNs.
- Investigate and present required platforms for developing robust UWSNs applications.
- Propose a thematic taxonomy to classify existing literature based on the most important parameters and comprehensively investigate recent advances solutions to get details concept and technical aspects.
- Highlight open research challenges of UWSNs as a guideline for future research to drive innovative development in various fields.

**Figure 1:**
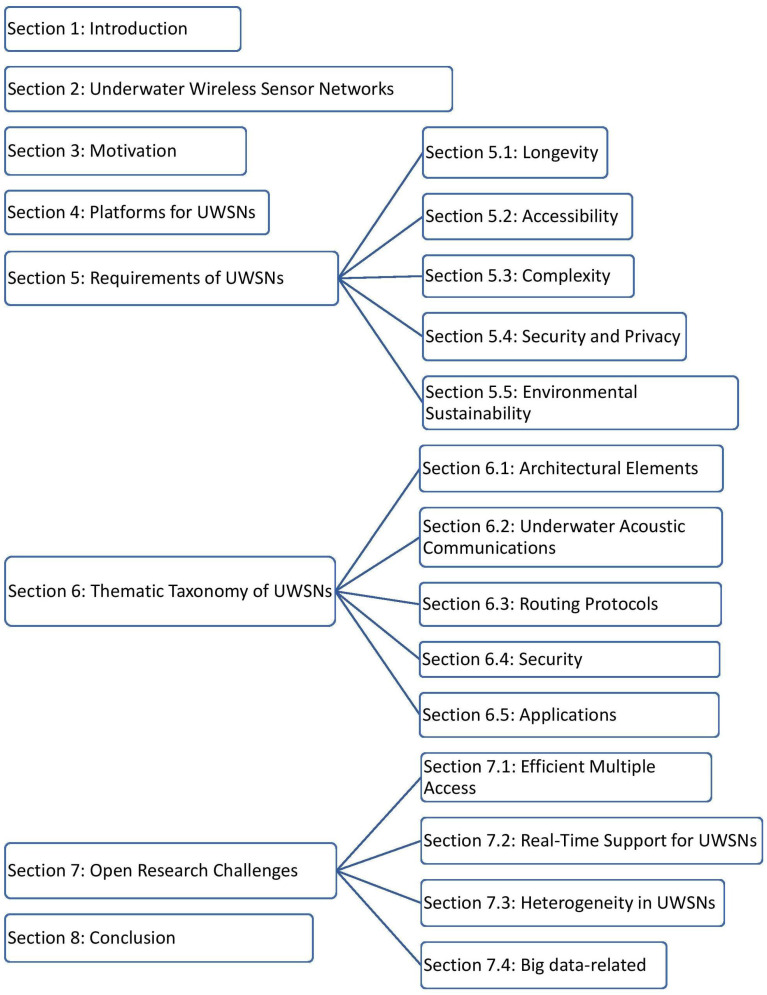
Overview of Research Work.

The paper is structured as follows: Section 2 briefly describes architecture and essential characteristics of underwater wireless sensor networks. Section 3 explains the research motivation and its significance for UWSNs. Section 4 discusses various platforms for developing UWSNs applications. Section 5 provides key requirements for implementing UWSNs. Section 6 compares, classifies and analyses the state-of-the-art of underwater wireless sensor networks with recent works. Section 7 details the research challenges in UWSNs. Finally, this paper concludes by summarising and emphasizing the future direction in Section 8. The paper is organized as illustrated in Figure 1. Abbreviations in this paper are defined in Table 2.

## 2. Underwater Wireless Sensor Networks

**Figure 2:**
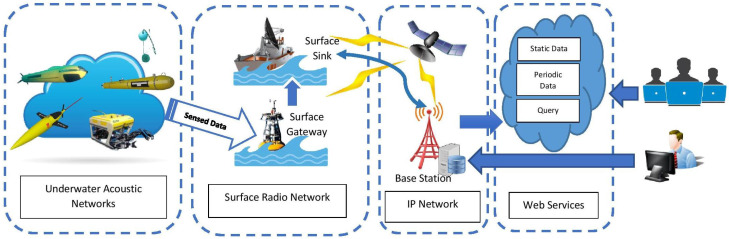
Overview of Underwater Wireless Sensor Networks.

The UWSN is a network used to perform monitoring of tasks over a specific region; it is equipped with smart sensors and vehicles that are adapted to communicate cooperatively through wireless connections [8]. The surface sink retrieves the data from sensor nodes. The sink node has a transceiver that can control acoustic signals received from underwater nodes. The transceiver also can transmit and receive long-range radio frequency signals for communication with the onshore station. The collected data are used locally or connected to another network for a particular purpose [9]. Figure 2 illustrates an overview of the UWSN environment. The network architecture incorporates traditional underwater wireless sensor networks designed by [8] and real-time underwater wireless sensor network architecture in the form of Internet of Underwater Things proposed by [10].

Underwater wireless sensor networks comprise of nodes that are deployable on the surface and under the water. All nodes need to communicate and exchange information with other nodes in the same network and with the base station. Communication systems in the sensor network involve the transmission of data using acoustic, electromagnetic, or optical wave media. Among these types of media, acoustic communication is the most popular and widely used method due to its attenuation features in water. The factor of low transmission is derived from absorption and conversion of energy into heat in water. Meanwhile, acoustic signals operate at low frequencies, which enables them to be transmitted and received over long distances.

## 3. Motivation

This section justifies motivation for the undertaken survey of underwater wireless sensor networks. The Underwater Wireless Sensor Network is of the developing technologies that is receiving significant attention and becoming a main focus of both researchers and practitioners [11]. With high technological advances in UWSNs, sensors have become smarter, smaller, and more flexible with lower power consumption, increased processing capacity, and the ability to operate in various underwater applications. Also, UWSN technology can be integrated with Internet Protocol-based systems in supporting the Internet of Things (IoT) and machine-to-machine (M2M) frameworks for real-time monitoring [12]. The rapid growth of UWSNs domain and the availability of modern sensor node technologies have forced the necessity to ensure that awareness is increasing every year due to their compatibility and broad application in various sectors [13,14]. According to [15], sensors and sensor networks require consistent research and exploration due to many unexplored resources, particularly in the oceans. Therefore, it is necessary for this study to provide insights and solutions with new technologies or a combination of existing works and technologies to ensure successful implementation of UWSNs. The requirements of UWSNs such as longevity, accessibility, complexity, security, and privacy and environmental sustainability (see Section 5) make UWSNs domain unique and challenging, especially for developers to come up with practical applications.

## 4. Platforms for UWSNs

**Figure 3:**
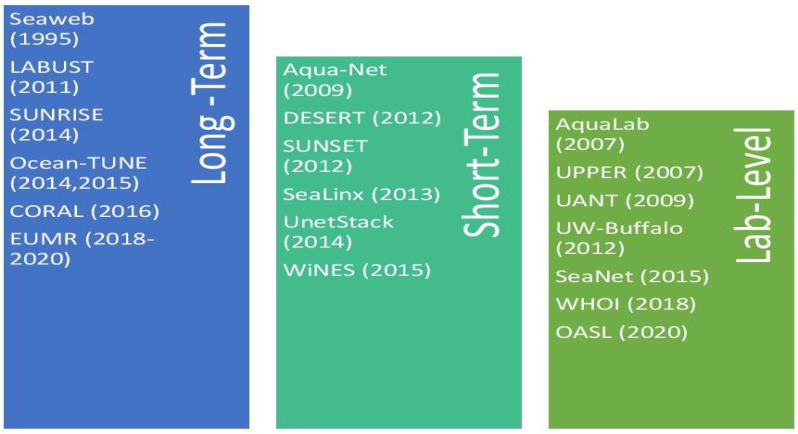
State-of-the-art Platforms in UWSN.

Over the years, research communities in UWSNs have developed various new designs and experiments in line with the technological advancements. According to [16], innovative solutions for evaluation, testing, and validation in UWSNs consist of three categories: software-based simulation, hardware-based simulation, and experimental field study. In comparing the three techniques, real-field experimental research, or testbeds, requires higher operating costs and presents difficulty in the data collection process and observations due to the unreliable environments of UWSNs. However, researchers typically need a combination of the techniques to verify the results of their experiment. Besides, mathematical models are used to support the verification and validation process. Figure 3 demonstrates the development of testbed platforms in UWSNs and their respective vital characteristics from 1995 to 2020. Generally, as categorized in [17], testbed platforms can be considered either long-term experimental, short-term experimental, or lab level.

## 5. Requirements of UWSNs

**Figure 4:**
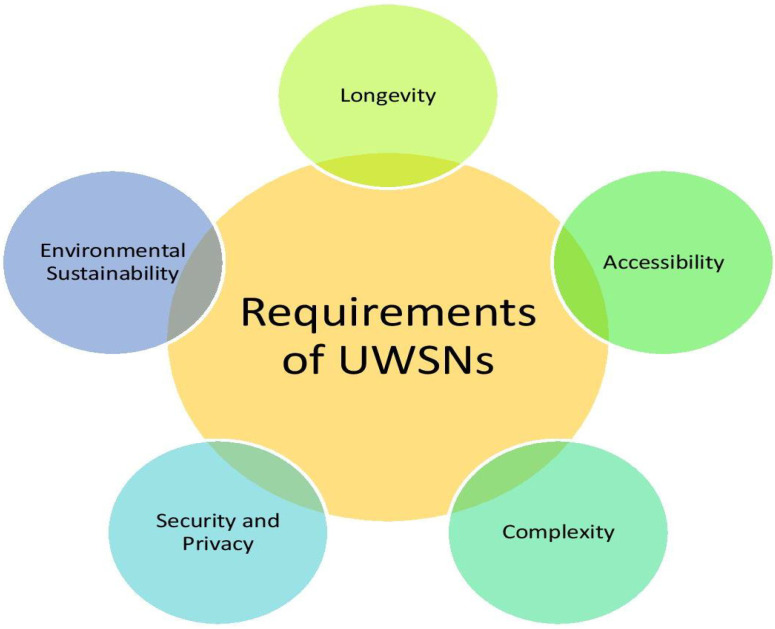
Requirements of UWSNs.

This section presents the essential requirements of UWSNs. Figure 4 represents these requirements.

### 5.1. Longevity

Network lifetime is one of the key requirements of UWSNs. It has a significance impact on the cost, time, maintenance tasks and performing underwater sensor nodes. It is crucial for maximizing the network lifetime, especially for mobile sensor nodes operations. Therefore, firmware has a vital responsibility in ensuring an effective practice of hardware features such as sleep modes, allows interruptions to replace polling and easy to set up. In addition, routing protocol and deployment of nodes have a huge role in controlling the energy consumption. It leads to a significant amount of research works on the development and evaluation processes.

### 5.2. Accessibility

Each sensor node communicates to each other within a communication range located in the region. The communication range is another important requirement for UWSN which affect the density of nodes, deployment feasibility and the network cost of the targeted monitoring area. There are two communication modes for underwater sensor networks; Acoustic and Optical communication. Underwater acoustic wireless communications have been one of the most used technology as it is accessible and requires communication over great distances. However, acoustic waves still have many shortcomings including scattering, excessive delay because of the low propagation velocities, high attenuation, low bandwidth, and adverse effects on the underwater creatures. Recently, orbital angular momentum has developed as an alternative multiplexing freedom to encrypt data onto vortex beams for enhancing the capacity of acoustic communication [18]. Due to the limitations of acoustic communication, another approach is to use optical waves. According to [19], the current research on underwater optical communications focus on expanding the data rate and transmission range. Optical waves have the advantage of higher data rate, low latency, and energy efficiency at the expense of limited communication ranges.

### 5.3. Complexity

The specification of sensor node placement at the position is also crucial for UWSN. Thus, a complexity factor need to be considered before setting up the networking platform which incorporates physical aspect, firmware and network configuration of nodes placement. Additionally, routing protocol selection and computing complexity contributes in identifying routes dynamically with no added information or prior knowledge about other nodes. Apart from that, node algorithm complexity is another factor that need to be considered since it influences the energy optimization of the nodes. Local nodes’ energy consumption is correspondingly depending on computational complexity and transmission power aspects. Underwater acoustic channel complexity such as multipath, Doppler shift, considerable attenuation and a high delay are also requirements that affect the performance of node localization methods.

### 5.4. Security and Privacy

UWSNs is correlated with security and privacy factors which related to sensor nodes connectivity, synchronization, and data transaction tasks. The dynamic features of underwater environment and its environment expose the network to various treats and malicious attacks. It is required for the networks to create trust before all the nodes can securely connect to the network to allow communication for information exchange. It is essential to study what level of security due to the increased computational load and the amount of transmitted data, yet consuming more energy within the network.

### 5.5. Environmental Sustainability

The deployment of communication technologies in UWSN is required to consider the impact to environment and wildlife. Ref. [20] reported that wildlife is influenced to ambient and boat noise which can lead to stress and the rise of extinction risk. Moreover, marine environment with increasing noise can generate behaviour changes, population distribution and hearing impairment of fish species.

## 6. Thematic Taxonomy of UWSNs

**Figure 5:**
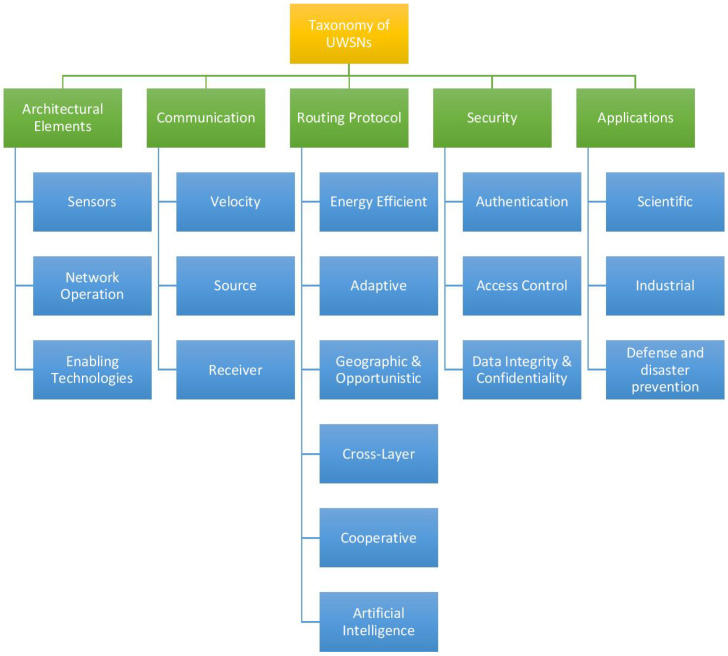
A Thematic Taxonomy of UWSN.

This paper proposes a taxonomy based on surveys and trend analysis of credible articles in the last five years. The most frequent topics discussed in the literature review is also taken into account before devising the thematic taxonomy. Figure 5 depicts a thematic taxonomy of UWSNs to better understand its characteristics. It categorizes the key attributes according to Architectural Elements; Communication; Routing Protocol and Standards; Security; and Applications. These attributes are discussed in the following sections:

### 6.1. Architectural Elements

UWSN architecture is classified based on the types, capabilities of the sensor nodes in the network, and spatial coverage of the applications.

#### 6.1.1. Sensors

A hybrid architecture consists of both static and mobile sensors. In a two-dimensional (2D) space, the static sensor node is generally mounted on the seabed and communicates with a sink node for data transmission through multi-hop communication within multiple clusters [5]. However, a static three-dimensional (3D) architecture has a slightly different setup, in which sensors are deployed according to different depths by adjusting the length of the wire connected to the anchor on the sea bottom, with the support of inflated buoys. In the mobile architecture, the sensor nodes can move freely, enabling the dynamic change of the network topology. The mobile node requires two transceivers to maximize network capabilities for data collection. It can consist of remotely operative underwater vehicles (ROVs), autonomous underwater vehicles (AUVs), or sea gliders. The third type is a hybrid architecture that combines static and mobile sensor nodes to perform specific tasks [21]. In a hybrid system, mobile nodes can act as a router or controller to communicate with static or standard sensors for data sensing.

UWSNs can improve monitoring and prediction activities, particularly on the ocean. For this reason, the coordination of technology and hardware is required to achieve the latter goal. Previous studies and experiments have shown that a combination of AUV and underwater sensors allow the implementation of observation or monitoring applications at different depth levels of water [8]. In [22], states that the integration between the sensor and AUV requires a network coordination algorithm to accomplish adaptive sampling and self-configuration. An adaptive sampling is a strategy to controll mobile vehicles and move them around the covered areas to perform the data collection process. Self-configuration is a procedure for AUV intervention to detect holes in the network due to the failure of sensor nodes or channel destruction. The synchronization between sensor nodes and AUVs requires obstruction avoidance via coordinated routing design.

#### 6.1.2. Network Operation

The objectives of network operation in underwater sensor networks are to manage and optimize various functions, characteristics, and performance criteria for appropriate applications. According to the recent articles published, we conclude that the trend of research and application development emphasizes more on localization and deployment tasks which has become the basis of underwater wireless network design to enhance overall network capabilities. Therefore, this section discusses the essential techniques and relevant features of each task to improve the network performance.

(A) Localization

Localization techniques have been widely explored in UWSNs and play a critical role for typical applications to provide information on the location of sensor nodes. In our review of published papers from 2013 to 2020, we categorized the localization algorithms into three main categories: stationary, mobile, and hybrid algorithms. The classification is subject to the movement of sensor nodes in UWSNs. Based on those categories, most of the researchers focused on localization techniques for stationary nodes.

For the stationary localization algorithm, all sensor nodes are fixed and static in the specific region of interest either attached to surface buoys or anchored on the sea floor. A variety of techniques are available for determining the location of stationary nodes. A recent method in [23] proposed the use of standard ray equations to manage the existence of ambiguity in the anchor node position based on the rigidity theory. Ray-bending is the leading property for estimating accurate positions of the nodes in their simulation. Previously, ref. [24] emphasized that optimizing sensors can significantly improve the performance of sensor localization techniques. The collected data is only essential if the position of the nodes is accurate. The authors applied a Cramer-Rao lower bound theory to obtain the optimal sensor position. Localization techniques also depend on packet transmission rates, especially for self-localization. For instance, ref. [25] proposed a method that aimed to reduce localization time with collision tolerance.

Some applications particularly monitoring activities in the ocean require mobile sensors to move freely underwater in performing these monitoring tasks. The algorithm for mobile sensor localization is divided into centralized and distributed algorithms. In the centralized algorithm, the sink node acts as a center point to calculate the location of nodes; in the distributed algorithm technique, each node performs localization tasks individually. In any research studies or UWSNs applications that involve mobile nodes, energy consumption is a crucial aspect that must be considered, as mobile nodes require high energy to move, and it is difficult to recharge or replace battery power in real applications. Some recent studies have been conducted, including [26], which proposed to reduce energy consumption by decreasing communication overhead in the data transmission process. Alternatively, ref. [27] recommended self-localization with free time-synchronization based on ranging optimization. The researchers found that the uncertain speed of the sound would cause the distance estimation of the node localization to be less accurate.

A hybrid localization algorithm is derived when both static and mobile nodes cooperate in executing a specific application. Typically, mobile nodes perform as data collector to gather data from static underwater sensor nodes. Instead of using only current location information in the estimation-based method, a prediction-based method computes node localization using both current and previous location information of sensor nodes.

(B) Deployment

Underwater wireless sensor networks comprise some nodes that are deployed underwater and some deployed on the surface of the water and accomplish their respective functions in the specific areas. Underwater sensor nodes that cover a sparse region are essential to be optimally deployed to use the limited battery life. Node deployment is considered to be a fundamental procedure in underwater sensor networks due to its capability to support many essential tasks, such as routing protocol, localization, and network topology, which contribute to significant impact to network performance. In [28], the node deployment in UWSNs is categorized into three groups: static or fixed, self-adjusted or limited mobility, and free mobility or movement-assisted deployment. In the static implementation, all sensor nodes are fixed in a particular region of interest and attached to surface buoys or anchored on the sea floor. This algorithm has higher coverage and connectivity compared to self-adjustment and mobile deployment but requires a more significant number of sensor nodes to cover a particular monitored area. For self-adjustment deployment, the movement of sensor nodes is generally in a vertical form with limited mobility. However, it is different from mobile deployment, where sensor nodes such as in an autonomous underwater vehicle (AUV) can move freely in all directions. Most of the mobile deployment algorithms in UWSNs have focused on reducing energy consumption as well as improving coverage and connectivity.

An underwater mobile sensor can change an initial node placement based on the newly obtained information of the target in real time. According to [29], a rearrangement in sensor node position is the strategy to accomplish a final anticipated configuration. Reorganization or redeployment is essential after some changes have occurred to the networks, which are typically caused by sensor failure (malfunction, energy reservation) or due to target/event detection. Based on previous studies ([30,31,32]), the authors expected that the mobile sensor nodes would be able to change the current position dynamically to provide maximum connectivity and maintain the network coverage. Furthermore, refs. [33,34] also emphasized that mobile sensor nodes can improve the detection rates compared to static and hybrid sensors. Table 3 shows the current algorithms that are garnering increased interest among researchers. Overall, most of the mobile deployment algorithms consider coverage, connectivity, and energy efficiency as the objectives.

#### 6.1.3. Enabling Technologies

There have been many recent advances in UWSNs, which have gone through rapid growth in various fields. Technological innovations in communication facilitate underwater sensor nodes and applications to interact with each other to provide services for users and adapt to varying requirements and preferences [45]. This innovation has become a driving force in Industry 4.0 that supports the deployment of Industry Internet of Things (IIoT). IIoT allows data to be forwarded securely to the cloud network from various sensor nodes and updated from time to time. According to [46,47], IIoT is the integration of industrial wireless networks and IoT technologies as a single system that consists of cloud networks, machines, equipment, and terminals. Consequently, recent developments in the field of IoT and UWSNs have led to a renewed interest in the Internet of Underwater Things (IoUT). IoUT allows a variety of underwater nodes to communicate with each other by collecting and transferring data to the surface station with high internet speeds. A study in [10] proposed an architecture of IoUT that consists of three layers: perception layer, network layer, and application layer.

**Figure 6:**
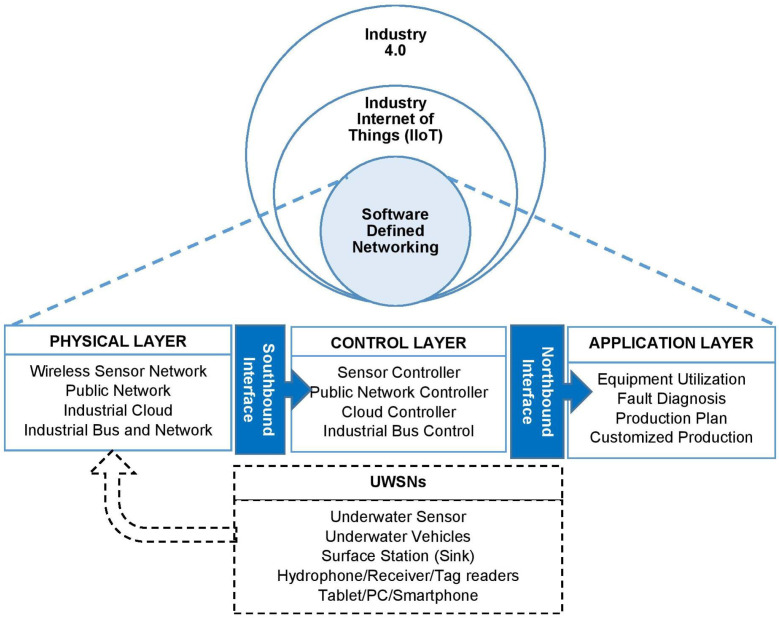
Relationship of SDN, IIoT, IoUT and Industry 4.0.

Remarkably, a Green IoUT was introduced as a new advancement to decrease the negative consequences of greenhouse gases on the environment [48]. Apart from this greenhouse effect, ref. [49] stated that underwater nodes and vehicles require high power consumption, which can stop the operation of critical missions or applications prematurely. This issue led the researchers in [50] to initiate emerging energy-efficient design for UWSNs, particularly for exploring offshore gas and oil environments. Within the next few years, underwater communication systems will confront some challenges in the integration of heterogeneous nodes or underwater vehicles, complex architectural design, and various underwater applications. For those reasons, ref. [51] introduced the next-generation network paradigm, known as Software Defined Networking (SDN), to support innovation practices, improve network flexibility, conduct efficient resource allocation, and control the operational cost. The architecture of SDN IIoT integrates three layers [47]. The sensor data from the physical layer moves to the control layer through a southbound interface and subsequently is transmitted to an application layer via a northbound interface and API. Figure 6 provides apparent relationship of emerging technologies among SDN, IIoT, IoUT, and Industry 4.0.

### 6.2. Underwater Acoustic Communications

The area of acoustic research and development has grown significantly over the past half-century, primarily in ocean acoustics. An acoustical approach is implemented commercially to detect submarines and even marine mammals. The military field is also parallel to commercial acoustics communication, particularly regarding those applications that relate to ocean surveillance. Consequently, this section discusses the main principles of underwater acoustic communication including sound velocity features, sources, and receivers. Additionally, all factors that affect the speed of sound and how it influences the performance of the system or network devices operation are also addressed.

#### 6.2.1. Sound Velocity

**Figure 7:**
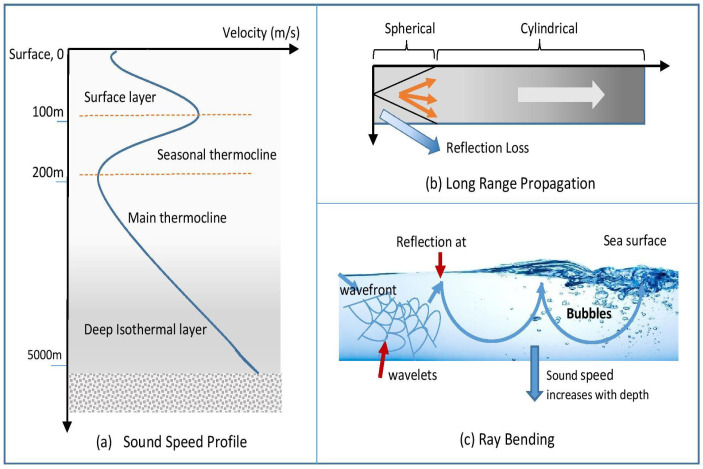
Sound Velocity Factors.

Acoustic waveforms in water are dependent on the sound velocity and the environment. According to [52], several key factors affect the sound velocity in water: temperature, salinity, and hydrostatic pressure through empirical experimentation. Figure 7 shows how these factors influence the velocity of sound in the ocean; key points of these factors are discussed below.

1. Temperature. The sound velocity and water temperature are closely related to each other: the velocity will be higher with an increase in water temperature. When approaching the surface of the water, the temperature increases as well as the sound velocity.
2. Salinity. The second factor that affects the velocity of sound in water is the salinity ratio. However, the salinity factor has a smaller effect on the velocity of sound compared to the temperature. Different concentrations of dissolved salts in pure water affect sound velocity. The level of ocean salinity is typically 35 p.s.u; this value varies depending on the characteristics of the water, and the effect of rock, soil, and atmosphere. Another factor regarding salinity levels is that they vary according to the depth of water.
3. Hydrostatic Pressure. The hydrostatic pressure factor has also effect on the velocity of sound in the water. Hydrostatic pressure increases the velocity of sound with depth [53]. The increase in depth is directly proportional to the increase in hydrostatic pressure.

##### Sound Velocity Profile

The ocean comprises two main regions based on ocean depth. Each level of depth produces different sound velocity variations, known as sound velocity profiles.

1. Ocean Depth below 200 m. The surface layer (0–100 m) is subject to change of environment, wind, and temperature. The wind circulation can mix up this layer and convert wind power to isothermal (mixed layer). The sound velocity is reduced dramatically if the wind speed is higher than 7 m/s due to the domination of bubbles found at a distance greater than 10 m below the surface of the water. In the seasonal thermocline region (100–200 m), the temperature changes seasonally. The temperature decreases according to the depth of the water. Consequently, in the winter season, the thermocline is weak since the surface of the water is continuously cool.
2. Ocean Depth above 200 m. At depths of 200–100 m, there is a region with minimal sound speed known as the main thermocline. At this depth, the water temperature begins to increase. In the deepest zone, known as the deep isothermal layer, the temperature characteristics depend on the density of water and water salinity. Nevertheless, the impact of hydrostatic pressure on the sound velocity is significantly higher compared to temperature and salinity.

##### Ray Bending

The sound rays interchange through the medium at a fixed rate, even with the variable speed of sound. The categorization of ray bending is either qualitative or quantitative. In qualitative ray bending, the sound speed will increase according to the depth in parallel with the increasing number of bubble populations. The population of bubbles decreases with increasing paths at the sea surface. When the acoustic energy concentrates within a layer at the sea surface, a reflection will occur. It does not spread in all directions, because it causes the speed of sound to be minimal where the course of the wavefronts propels toward the water depth. This velocity profile is known as the SOFAR (Sound Fixing and Ranging) channel. Conversely, in quantitative ray bending, a sound ray moves horizontally through the points, and the sound velocity increases linearly, in parallel with the depth.

##### Long Range Propagation

The reduction of sound in signal-to-noise amplitude due to long-range propagation, especially in the SOFAR channel, is influenced by geometry, attenuation, and thermometry. According to the inverse-square law, acoustic wave strength decreases when a wave with spherical symmetry geometrically deviates from the primary source point. Nevertheless, in the SOFAR channel, the action is relatively different. The rays do not bend spherically but spread from a line source in the form of a cylinder symmetry. The inverse-square law concludes that geometric distribution can reduce the strength of acoustic waves in parallel with decreasing distance.

There is a significant difference in the acoustic absorption rate between seawater and pure water within the frequency range of 5–50 kHz. The difference in frequency occurs due to a mechanism associated with viscosity under 100 kHz, where the element of magnesium sulfate is loose. As highlighted previously, water temperature is one of the main factors affecting sound velocity. At a depth of 1 km in the ocean, temperature and the sound speed increase when sound spread over distances with 4.6 m/s per degree centigrade.

In conclusion, both frequency-dependent absorption and noise and low and variable propagation speed have a significant impact on the design of underwater communication networks. Different scenario characteristics and even current different time spots lead to different channel responses. As a consequence of the long propagation delays of underwater transmission, the networks also be affected of space uncertainty, and spatial inequity [54]. As the packet reception time depends on the distance to the transmitter, the channel becomes free first at the transmitter and later on at the receiver.

##### Sea Surface

The parameters that affect the sound velocity in different areas such as at the boundary, the bottom and the interface of the ocean provide various proportions. The composition and density of hard rocks and sediments at the sea bottom are factors that affect the increment or decrement of sound velocity. Furthermore, the bubble population at the sea surface is another factor that influences the velocity of sound. The presence of bubbles causes the average density of water to rise. The velocity of sound reduces the occurrence of bubbles, as shown by formulations and experiments.

#### 6.2.2. Sound Sources

As demonstrated in [55], primary underwater acoustic sources derive from natural and anthropogenic sources that are generated from ambient noise spectra. Besides, the underwater noise level is influenced by wind speed at different frequencies and the sea environment (the presence or absence of sound sources from nearby ships and marine life) [56]. As indicated in the Wenz curves [55], the spectral state of the sea is between 200 Hz and 500 Hz. Apart from ambient noise spectra, anthropogenic sounds are another primary source of underwater noise. All of the sound sources (e.g., shipping sound exposure, sonar, volcanic eruptions, and marine seismic) have a negative impact on noise pollution to the marine life, including hearing loss and behavioral changes.

#### 6.2.3. Sound Receiver

A sound receiver is an essential device in underwater acoustic applications and consists of a transducer, amplifiers, and data acquisition boards. An underwater sound transducer is a device that converts acoustic energy to electrical energy (hydrophone) or vice versa (projector). Consequently, an amplifier can be connected to the hydrophone to increase the amplitude of the sound, which can then be measured using a voltmeter. The outputs in the analog wave can be converted into digital signals using a data acquisition system or, vice versa; digital signals can be turned into analog signals for processing purposes. A Data Acquisition (DAQ) board consists of an analog-to-digital converter (ADC), TTL level logic inputs, onboard timer, and digital-to-analog converters (DAC).

In carrying out the tasks of underwater wireless sensor networks, real implementation is usually quite challenging due to the environmental factors especially in deep sea and also high operating costs. Therefore, most studies are currently using simulation as another alternative. In conducting the simulation for UWSN, the conditions and environmental properties give a significant impact on the performance system.

Table 4 lists some recent studies that take into account all the factors including the environment, and system parameters in obtaining the results of the study. Methods, advantages, and description of the study are reported for further review.

### 6.3. Routing Protocol

Routing protocol becomes a crucial design task in a network layer to provide various requirements for acoustic communication to identify and sustain the network routes. In the past, as well as the present, many protocols have been proposed and explored to enhance network performance for underwater sensor networks. The authors have analyzed the recent study of routing protocols in UWSNs and identified that the principal objective of most routing protocols is energy efficiency (see Table 5). The primary challenge of operating underwater sensor nodes is to maintain the limited amount of energy to continue operation.

Acoustic underwater communications consume a larger value of energy compared to the terrestrial radio frequency. Those sensors located which are one-hop away from a static sink would suffer from a severe exhaustion of their battery power, which may cause energy holes. It may result in possible network disconnection and consequently preventing messages from achieving the sink. The unique characteristics of underwater environment should be taken into consideration through the operation of the time-varying channel in designing routing protocol. Most of the early work on routing layer has concentrated on compromising with great delays while producing energy efficient communication but has not included important propagation factors such as frequency-dependant attenuation, bottom surface reflections and Doppler effect, which highly affect energy consumption through both power and rate.

On top of that, the current routing protocols also emphasize the use of adaptive routing, opportunistic, cross-layer, cooperative, and artificial intelligence-related routing protocol to satisfy different requirements of UWSNs (see Table 6). Naturally, the underwater environment is harsh, unreliable, and sparse. In consequence, these unstable states expose UWSNs to natural fragmentation due to the mobility of sensors, thereby affecting the reliability of data transmission from source to destination. Therefore, the designs and techniques of routing protocol are required to deal with these issues.

### 6.4. Security

UWSN sensor nodes are typically sparsely deployed in harsh and hostile environments, which makes them highly vulnerable to various types of network attacks. Therefore, security is an essential aspect to consider to ensure that the operation of an application runs smoothly and provides reliable data. The features of UWSNs themselves (e.g., high propagation delay, limited bandwidth, high bit error rates, and computational capability restriction) have also exposed them to internal and external attacks.

#### 6.4.1. Authentication

The preliminary phase for an UWSN is bootstrapping, in which a sensor alerts to be linked to the network. Each sensor node (as a receiver) is required to authenticate the sender for verification and identification purposes before receiving the message. All nodes connected to the network need to have permission or authorization from the network services during transmission. After the authentication process successful, the nodes will ready to execute any tasks assigned according to encoded procedures. Thus, execution of a robust authorization technique is essential in UWSN. Several security requirements are identified in [96] to protect a wireless sensor network as well as UWSNs from internal and external attacks.

#### 6.4.2. Access Control

In access control process, the restriction of data access is applied to secure front-end and back-end data, services and resources of UWSNs. The source of risk such as malicious node and unauthorized data vulnerability can be avoided or decreased by employing smart devices or adaptive method. There are two types of access methods; centralized and distributed techniques. All the inquiries of access control for centralized approach must go through a server to authorize connection. However, in distributed access control method, access control server designates an entity to allow access to UWSNs recources. The network services should always be available in the system to overcome any possibilities for communication errors in UWSNs.

#### 6.4.3. Data Integrity and Confidentiality

Additionally, another crucial security requirement is integrity. Each node has to preserve the confidentiality of the data during data transmission. Apart from the data, the packet head also must be encrypted to protect the identity of each node. Moreover, the message must be fresh. The node can ensure that old information from previous transmissions is not transmitted or received by implementing the time-varying technique. If the node has previously sent data, that particular node cannot deny that transmission which was performed by itself. This requirement is known as Nonrepudiation.

Several studies on identifying existing forms of attack were conducted. Based on the findings, the invasion in UWSNs occurred during data transmission, in the form of denial-of-service (DoS), assaults on physical nodes, replication, and impersonation. According to [97], attacks of DoS often occur in UWSNs due to the high level of effectiveness of DoS attacks, difficulty in detection, and low costs of operation. The analysis of DoS attacks on the physical layer is discussed in detail by [98] and [99]. They tested the results in real test implementation. An attack on data is also a common security issue in UWSNs. Data management using information-centric architecture is one of the methods to protect the data from DoS attacks. However, strikes from smart DoS types are still capable of affecting the data. In consequence, ref. [100] incorporated the concept of machine learning in information-centric architecture to detect various types of mobile attackers.

Based on previous research findings, security issues in UWSNs are primarily focused on security in localization, routing, data aggregation, trust model, and intrusion detection. Five processes proposed in [101] protect location by integrating the trust model in the evaluation process to further enhance the security features of the localization technique. The application of this trust model was improved by [102] in implementing centralized trust management in UWSNs using a cloud model. The objective of the trust management method is to determine the trustworthiness of each sensor node by applying a mathematical approach to obtaining trust evidence. In [103], the researchers implemented several experiments and found that an efficient encryption algorithm can preserve the confidentiality and integrity of the data. Additionally, the approach can also reduce overhead communication in the application layer. After the node deployment process, key generation based on RSS will allow pair nodes to update the secret keys at any time. A wireless sensor network uses the technique commonly, but the method is not appropriate for the harsh and inconsistent environment of UWSNs. Therefore, ref. [104] introduced a crucial multi-channel generation algorithm to produce more efficient secret bits to allow secure acoustic communications in UWSNs.

### 6.5. Applications

**Figure 8:**
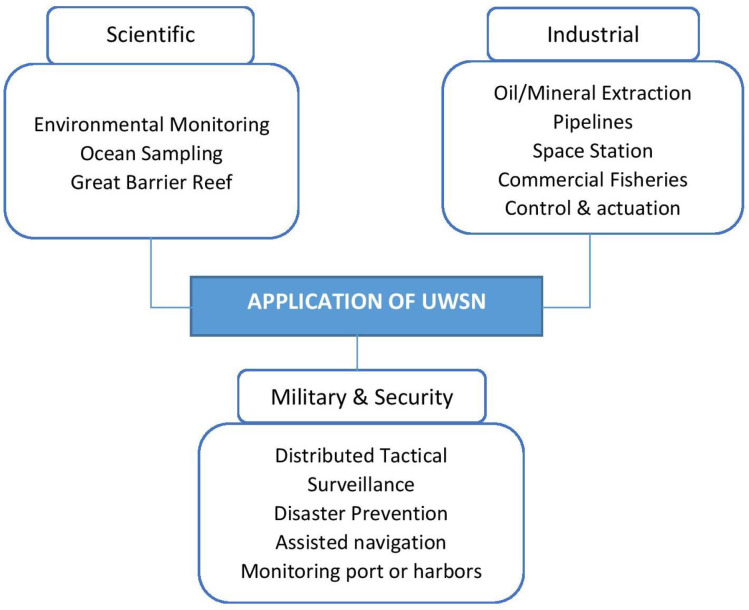
Applications of UWSNs.

UWSN technology can replace traditional approaches by offering real-time monitoring, an onshore system to control underwater appliances remotely, and advanced devices for data recording. Commonly, UWSN applications are composed of three categories: scientific, industrial, and military and security (see Figure 8). In the military, sensor nodes are used to detect the movement of enemies and their location. It can be applied to monitor ports and harbors, conduct border surveillance, identify underwater mine locations, and detect enemy submarines. In the case of natural disasters, sensor nodes can detect marine environments by performing seismic monitoring in advance of disasters. A wide range of applications requires rapid developments in standards and technologies to support and enhance the growth of new applications. While there are many different applications, this section presents a survey of recent developments in the domain of UWSN applications, that are assisting in scientific, industrial, and defense and disaster prevention activities.

#### 6.5.1. Scientific

A wide range of applications for underwater wireless sensor networks in the scientific field is classified as environmental monitoring, ocean sampling, and, particularly, Great Barrier Reef activities. The environmental monitoring application serves to monitor the amount of pollution such as chemical and biological that is deposited on the seabed [2,8] and to conduct water quality observation that involves participation from people who are affected by using real-time notifications [45]. Moreover, in [12], robotic fish have been employed to measure oxygen levels in water as well as for pressure and temperature monitoring [70]. An ocean sampling application reported in [17] focuses on monitoring a large coastal area to study the ocean phenomena by applying underwater sensor vehicle technology at several locations. The collected data will be sent to the shore automatically for further observations. In [21], the researchers present a coral reefs application that combines the technology of the sensor network, big data, and the Internet of Things (IoT) to study the effects of ocean salinity, temperature, humidity, and pressure on coral bleaching and marine ecosystems. Long-term marine environmental monitoring can also be implemented using a combination of different types of agents and communication. The research team [105] conducted experiments at Biograd Na Moru, Croatia by combining Autonomous Surface Vehicles (ASVs), highly mobile artificial fish, and artificial mussels for data collection purposes.

#### 6.5.2. Industrial

Industrial applications in UWSNs lead to a significant impact in facilitating commercial activities. UWSN has potential to monitor underwater oil and gas pipeline monitoring applications. The authors in [106] have designed a prototype for monitoring oil and gas in pipeline underwater. The system was developed to provide statistics reporting regarding pipeline health that connected over large areas. In [107,108], the authors also designed an underwater oil and gas pipeline monitoring system that require control of an actuation component.

Fish farming is one of the most demanding production that contribute to a good economic resources. However, it requires a strict monitoring system to monitor the habitat conditions of the fish. The authors of [109] developed a Zigbee-based UWSN monitoring for large fish farms and can be accessed remotely by interested user. Besides, the system has the ability to monitor the fish farms based on dissolved oxygen, pHvalues, temperature, the water level, and humidity parameters. Additionally, wireless cameras are used and integrated with the system and the Internet for wireless monitoring anytime and anywhere in the world. In addition, authors of [110,111] also developed similar commercial fishery monitoring system by using acoustic waves as the communication media.

#### 6.5.3. Defense and Disaster Prevention Application

Military and defense applications use a combination of underwater sensors to identify potential enemies earlier by conducting ports and harbor monitoring and control [112], sea mine detection [113], border protection from illegal battleships or submarines, and distributed tactical surveillance [114]. Furthermore, UWSN advanced technologies such as the mobile underwater sensor network are also used to provide early warnings of natural catastrophes such as seismic activities on the seafloor [112]. Jain and Virmani [115] designed a model for real-time tsunami prediction and used data collected from the occurrence of the tsunami that hit the Indian Ocean in 2004 for evaluation.

Table 7 shows the implementation of recent studies in various networks and settings. Overall, communication between nodes is achieved using acoustic waves or a combination of both radio frequency waves and acoustics. The network settings are constructed based on the type of application, region, network size, water depth, communication type and frequency, distance between nodes, type of sensor and the total of nodes.

## 7. Open Research Challenges in UWSNs

**Figure 9:**
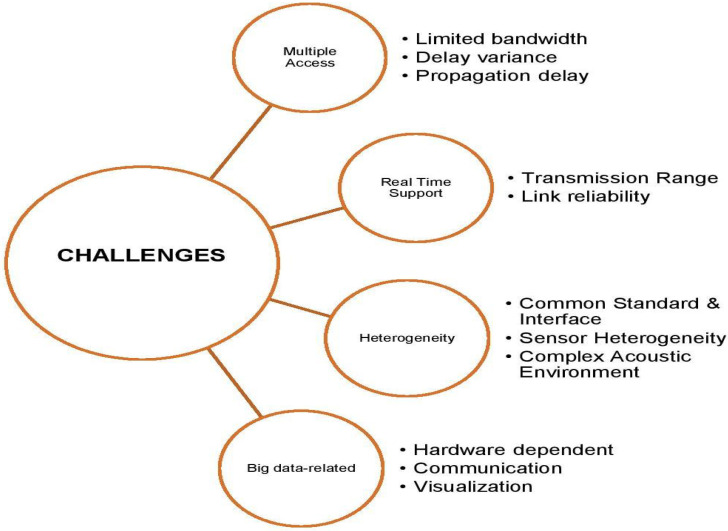
Open Research Challenges in UWSN.

Research and implementation of UWSNs have been growing and widely applied in both research and industry. However, after reviewing the current trends and studies, several challenges remain to be addressed for further development. This section briefly explains the current challenges experienced with UWSNs as shown in Figure 9.

### 7.1. Efficient Multiple Access

Communication is a vital element in UWSNs and enables collaboration among sensor nodes and interactions between the node and sink to perform data transmission/exchange, location and configuration processes.

1. Limited Bandwidth

According to [130], factors such as noise, multiple paths, path loss, and Doppler spread can affect UWSN communications and lead to a limitation of channel bandwidth. Although many studies addressed communication based on simulation analysis and real experiments, there are many more challenges and concerns that need to be resolved in future, particularly MAC protocol in the data link layer. The objective of the medium access control protocol (MAC) is to coordinate all nodes in the UWSN network to access shared channels. It is also to ensure that the data sent is valid and that it reaches the destination efficiently.

2. Delay Variance

Delay variance is an element that causes inaccurate estimates of round-trip time (RTT) values and presents difficulty in determining the waiting time in the MAC protocol. However, ref. [131] found that most of the studies on MAC protocols did not consider variance delay in their solutions.

3. Propagation Delay

Commonly, the acoustic signal does not propagate well underwater and is five times lower than electromagnetic waves in the air. The propagation delay is the critical challenge, particularly in the MAC protocol solution. The waiting time for MAC or retransmission time-out (RTO) has a direct impact on throughput. In [131], the authors found that the existing fixed RTO is inefficient. Furthermore, due to the long propagation delay in UWSNs, a handshaking process, which enables channel sharing among all nodes, results in high costs compared to the terrestrial sensor network. It will eventually lead to handshaking overheads that cause low bandwidth.

### 7.2. Real-Time Support for UWSNs

Critical applications such as natural disaster detection (e.g., earthquakes and tsunamis), oil spills, and territory surveillance require real-time support via an early warning to the authorities before executing necessary actions. However, in supporting the implementation of real-time UWSNs, researchers must consider and overcome the existing challenges.

1. Transmission Range

One of the unique features of the underwater environment is the ability of signal to be absorbed, based on the water depth level. The frequency should be reduced to minimize the effect of signal absorption. However, the range of transmission will become longer and, consequently, challenges arise concerning the probability of interruption and high data collisions.

2. Link Reliability

Link reliability is another vital element to achieve high delivery rates in real-time scenarios. The link reliability factor among the sensor nodes in the network can influence the delivery rates and consequently affect the transmission loss, which reduces the accumulated intensity of the waveform energy to propagate from source to destination. The presence of noise in the underwater environment can also cause a lost transmission, which consequently affects the reliability of data transmission. Furthermore, the reliability of connection may result in repeated data retransmission that increases the energy consumption of nodes and bandwidth usage. Therefore, a consideration of data transmission efficiency is crucial to avoid untrusted links.

### 7.3. Heterogeneity in UWSNs

Adequate integration and communication among underwater vehicles and sensor nodes are essential to support a wide range of emerging applications. The homogeneous sensor network is unfavorable for many applications.

1. Common Standard and Interface

It becomes a crucial challenge for the cooperation of heterogeneous nodes, and optical and acoustic modems in an operational setting because of the lack of a common standard and interface to support communication and message exchange.

2. Sensor Heterogeneity

Besides the different type of assets, sensor heterogeneity that enables cooperation of both mobile and static nodes in implementing specific applications also faces another challenge. The challenge arises in nodes communication holes to maximize data transmission.

3. Complex Acoustic Environment

Additionally, the problems of heterogeneous underwater sensors emerge from the complex acoustic environment, particularly in shallow water. The detection of acoustic signals is becoming increasingly difficult with the existence of multiple control techniques from heterogeneous nodes as well as the challenges from the unique features of UWSNs themselves.

### 7.4. Big Data-Related

Integrating UWSNs and big data is one of the recent trends. However, the flexibility and scalability of a traditional UWSN is still dissatisfied because of its dependent ability on hardware infrastructure. In UWSN, big data processing encounters several challenges related to accuracy, real-time analytics, and visualization.

1. Hardware Dependent

Underwater sensor nodes such as autonomous underwater vehicles (AUVs), wheels, or unmanned aircraft consume energy from a battery and difficult to reconfigure after deployment. Hence, it is a challenge for another system to do customization due to different data format, protocol, and service constraint of varied applications.

2. Communication

Underwater sensor nodes are commonly coming from different manufacturers. As a result, it makes them challenging to perform communication as cooperation among UWSN technologies is not flexible to each other.

3. Visualization

The result analysis of the UWSN application is significant and understandable if there is a synchronization between visualization and data analytics. However, it is a challenge to establish a visual representation of highly heterogeneous big data.

## 8. Conclusions and Future Remarks

A unique design and characteristics of underwater sensor networks provide advantages in the development of scientific, industrial, defense, and disaster prevention applications, which are dissimilar with terrestrial sensor networks. However, the features of UWSNs and application requirements restrict their use and reveal the gap between applications and technologies. This study shows the essence of acoustic communication as an elementary principal for designing and executing the algorithms, protocols, and services in UWSNs to manage such limitations. First, we investigated the state-of-the-arts in the literature related to UWSNs. We then determined the requirements of UWSN, and provided a thematic taxonomy. This paper analyses the literature from trusted and well-known article databases. Investigation of these classifications assist us to identify the challenges for future improvement and open more opportunities for long-term success in the field of underwater sensor networks.

Even though UWSNs have received a great number of improvements in the previous few years, there is still substantial room for improvement, especially in implementing systems on a large scale. As future work, the researchers can offer better solutions on node mobility with high monitoring area (with high neighbourhood range) scenarios to investigate the effect on network connectivity, coverage, energy consumption and network lifetime. To increase efficiencies of the UWSNs and improve its performance, the studies should direct the focus of the prospective research towards implementing cooperative control among a few underwater vehicles. The future studies should enhance the cooperative communication of the vehicles in terms of channel bandwidth and autonomy level (such as covering a re-planning decision). Simultaneously, the subsequent studies could analyze the environment and underwater vehicles models to enable the algorithm to have a wider range of applications. Next, the researchers can plan to develop the significant high-level planning layer to designate the desired configurations or strategic region of interest that the vehicle ought to explore towards. The works could also cover more complex network scenarios including mobility, shadowing, multi-path fading and evaluate how it influences the results. Finally, a hybrid harvesting energy strategy for ocean environment monitoring needs to be taken into consideration, to harvest and apply more reliable renewable energies in the hostile marine environment. 

## Figures and Tables

**Table 1:** Comparison of the Proposed Paper with the Existing Surveys in UWSN.

Contributions	Previous Study					Proposed Study
	[3]	[4]	[5]	[6]	[7]	
Underwater Sensor Networks Architecture	***✔***	***✔***	***✔***	***✔***	***✘***	***✔***
Platforms for UWSNs	***✘***	***✘***	***✔***	***✘***	***✔***	***✔***
Requirements of UWSNs	***✘***	***✘***	***✘***	***✘***	***✘***	***✔***
Thematic Taxonomy of UWSNs	***✘***	***✘***	***✘***	***✘***	***✘***	***✔***
Architectural Elements	***✘***	***✔***	***✔***	***✔***	***✘***	***✔***
Underwater Acoustic Communications	***✔***	***✔***	***✔***	***✔***	***✔***	***✔***
Routing Protocols	***✔***	***✘***	***✘***	***✘***	***✔***	***✔***
Security	***✘***	***✘***	***✘***	***✘***	***✘***	***✔***
Applications	***✘***	***✔***	***✔***	***✘***	***✘***	***✔***
Open Research Challenges	***✔***	***✔***	***✔***	***✔***	***✔***	***✔***

**Table 2:** Key to Abbreviations used in the Paper.

Abbreviation	Description
ADC	Analog-to-Digital
API	Application Programming Interface
AUVs	Autonomous Underwater Vehicles
DAC	Digital-to- Analog
DAQ	Data Acquisition
IIoT	Industry Internet of Things
IoT	Internet of Things
IoUT	Internet of Underwater Things
M2M	Machine-to-Machine
Mbps	Megabits per second
ROVs	Remotely Operative Underwater Vehicles
SDN	Software Define Networking
SOFAR	Sound Fixing and Ranging
TTL	Time to live
UWSNs	Underwater Wireless Sensor Networks
WSN	Wireless Sensor Networks

**Table 3:** Mobile Deployment Algorithm in UWSNs.

Author	Algorithm	Objective	Deployment Criteria		
			Energy Consumption	Coverage	Connectivity
[35]	Self-deployment Particle swarm	Optimize events coverage	Yes	Yes	Yes
[36]	Construction of initial infrastructure	Node placement strategy to minimize transmission cost	No	Yes	Yes
[37]	Uneven cluster deployment	Improves network reliability and prolongs network lifetime	Yes	Yes	Yes
[38]	Fisher information matrix (FIM)	Target positioning precision	Yes	No	No
[39]	A three-dimensional coverage pattern and deployment scheme	Preserve network coverage	Yes	Yes	Yes
[32]	Game theory	Optimize mobility of nodes and targets	No	Yes	Yes
[40]	Integration of a realistic model and gradient descent method	Improve sensor node placement	Yes	Yes	No
[41]	Multiobjective optimization framework	optimal deployment of a sparse network of sensors against moving targets	No	Yes	Yes
[42]	Autonomous deployment algorithm for k-barrier coverage	Utilize self-deployment method to improve coverage	Yes	Yes	Yes
[43]	Greedy Iterative Approach (GFCND)	Improve network connectivity and coverage	Yes	Yes	Yes
[44]	Stratified Connected Tree	Optimize leaf nodes position to improve coverage and connectivity	Yes	Yes	Yes

**Table 4:** Several Related Papers that Deal with Environmental Parameters through Simulation.

Author	Method	Description	Environmental Parameters	System-Parameter	Advantages
[57]	Relative positioning system	the propagation times of acoustic used to measure the position of buoys	temperature, depth, salinity, bottom and water densities, wind speed or sound speed at bottom material	signal frequency, hydrophones’ depth, the aperture angle of the transducer or the position of the buoys	Able to understand the region surface current
[58]	Adaptation of data and model-based framework	apply a high-fidelity acoustic modeling infrastructure	Sea state, Sea floor depth	Source and receiver (Buoy) depth and speed, Source Level, Frequency (carrier), Bandwidth, Modulation	A set of behaviors able to extend the decision of typical behavior-based systems
[59]	An intelligent online framework for communication environment changes	Provide database tracks for communications layer visibility	Bathmetry, bottom type, water column	Mission Path Size, Ambient Noise, Sound Speed Profile, Vehicle Type	can provide acoustic modem optimization for collaborative AUV missions
[60]	A C-SLAM algorithm	communication packets generation with observed features	Doppler velocity	Strategy, design measurements	Allow associating the uncertainties position of vehicles without infrastructure
[61]	A decentralized formation control algorithm	Maintain the distance and angle without relies on leader robot information	location of the obstacle	avoidance layer, formation generation layer	Enable shortening the procedure of the information process
[62]	A data-driven method	Minimize the target location error of the onboard tracker	sound speed, noise level, reflection loss gradient, maximum depth	prediction steps, step time length, heading choices, maximum heading change decisions	ability to handle outliers and computational limitations
[63]	A software/hardware hybrid system	Real-time AUVs operation with acoustic modem telemetry	Ocean model, acoustic model	Communication model	The design is flexible to existing and new modems
[64]	stochastic level-set partial differential equations	calculate stochastic reliability in three different scenarios	Wind stress, ocean flows	vehicle-speed	the vehicles can move in unreliable flows of coastal ocean

**Table 5:** List of Current Techniques for Energy Efficient Routing Protocols in UWSNs.

Energy Efficient Protocol	Methodology	Advantages	Requirements	Performance
Joint Routing and Energy Management [65]	Minimize nodes communication energy throughout data transmission process	Balance energy distribution of all nodes	Next hops address, node capacity and low energy data transmission	Fair
DRP [66]	Find a path with high energy and transmission rate	Prolongs network lifetime, improve throughput	Periodic broadcast of HELLO packets	High
E-CBCCP [67]	Consider energy of the cluster heads	Reduce nodes communication cost and high network lifetime	Ocean environment is stable	Fair
EBET & EEBET [68]	Selection of high energy node	Practical for large scale network	Predetermined location of sensor nodes	Fair
E-CARP [69]	Allows the previous collected data to be stored at the sink node	Effective communication cost; Minimize energy consumption	Predefined location of both sensor and sink nodes	High
EBECRP [70]	Exploit the use of mobile sinks	Prolong network lifetime by reducing number of data transmissions	Sinks have knowledge of sparse and dense regions	Fair
SEEC, CSEEC & CDSEEC [71]	Perform clustering and the used of sink mobility	Minimize the energy consumption of sparse regions	Depth threshold of each node is 25 m	Fair

**Table 6:** Category of Routing Protocols Based on Features

Category	Protocol	Void Avoidance	Improve Data Delivery Ratio	Energy Efficiency	Multi Hop	Mobile /Static Nodes	Multiple/Single Sink	Location is Known	Cluster or Single Entity
Adaptive	SACRP [72]	No	***✔***	***✔***	Yes	Static	Single	Yes	Single
	AHH-VBF [73]	No	***✔***	***✔***	No	Both	Single	Yes	Single
	iAMCTD [74]	No	***✔***	***✔***	No	Mobile	Multiple	No	Single
	AVN-AHH-VBF [75]	Yes	***✔***	***✔***	Yes	Static	Single	Yes	Single
	QL-EEBDG [76]	No	***✔***	***✔***	No	Static	Multiple	Yes	Single
Geographic & Opportunistic	EnOR [77]	No	***✔***	***✔***	Yes	Static	Single	Yes	Single
	Co-improved Hydrocast [78]	Yes	***✔***	***✔***	No	Static	Multiple	Yes	Single
	VHGOR [79]	Yes	***✔***	***✔***	No	Static	Single	Yes	Single
	GEDAR [80]	Yes	***✔***	***✔***	Yes	Mobile	Single	Yes	Cluster
	GGFGD & GFGD [81]	No	***✘***	***✔***	Yes	Static	Single	Yes	Single
	3DRanDomProb [82]	No	***✔***	***✘***	Yes	Mobile	Single	No	Single
Cross-Layer	cross-layer protocol stack [83]	No	***✔***	***✔***	Yes	Static	Single	Yes	Cluster
	NCRP [84]	Yes	***✔***	***✔***	Yes	Static	Single	Yes	Cluster
	VBF-improve [85]	No	***✘***	***✔***	No	Mobile	Single	Yes	Single
Cooperative	Co-UWSN [86]	Yes	***✔***	***✔***	Yes	Mobile	Multiple	Yes	Single
	NC [87]	No	***✘***	***✔***	Yes	Static	Multiple	No	Single
	S-DCC [88]	No	***✘***	***✔***	Yes	Static	Multiple	No	Single
	HAMA [89]	Yes	***✘***	***✔***	Yes	Mobile	Single	Yes	Cluster
	CoDBR [90]	No	***✘***	***✔***	Yes	Mobile	Multiple	Yes	Cluster
	EOCA [91]	No	***✔***	***✔***	Yes	Mobile	Single	Yes	Cluster
	SPARCO [92]	No	***✔***	***✔***	Yes	Mobile	Single	Yes	Cluster
Artificial Intelligence Related	QKS [93]	No	***✘***	***✔***	No	Mobile	Single	Yes	Cluster
	QELAR [94]	No	***✘***	***✔***	Yes	Mobile	Single	Yes	Single
	UW-ALOHA-Q [95]	No	***✔***	***✔***	Yes	Static	Multiple	Yes	Single

**Table 7:** Comparison of Various Underwater Wireless Sensor Networks.

Reference	Application	Network Deployment			Communication		Sensor Node		
		Salinity Level	Network size	Operable Depth	Channel Frequency	Type	Type	Distance	Number
[116]	Fish farm	Ocean	Up to 2.4 km	30 m	26.8 kHz	RF, Acoustic	Static	6 m	5
[117]	River Monitoring	River	5000 m × 200 m	50 m	35 kHz	Acoustic	Mobile	300 m	2
[118]	Ocean Monitoring	Shallow Water	90 × 38 × 45 cm	Up to 3 m	433 MHz	RF, Acoustic	Static	15 cm	2
[119]	Environmental Monitoring	Sea	Up to 2 km	2 m	28 kHz	RF, Acoustic	Static	100 m	3
[120]	Water Quality	Sea	4500 to 5500 m^3^	45 m	25 to 40 KHz	RF, Acoustic	Static	110 m	3
[121]	Surveillance	Sea	23 km × 30 km × 300 m	50 m	1 kHz to 4 kHz	Acoustic	Mobile	75 m	2
[122]	Target Tracking	Sea	Up to 1 km	32 m	2 kHz	Acoustic	Mobile	300 m	2
[123]	Exploration	Sea	14.5 m × 12 m	2 m	2 kHz	Acoustic	Mobile	10 m	3
[124]	Survey Planning	Sea	600 m × 600 m	20 m	1 kHz to 4 kHz	Acoustic	Mobile	10 m	2
[125]	Target Tracking	Sea	30 m × 30 m × 25 m	25 m	2 kHz	Acoustic	Mobile	4 m	6
[126]	Surveillance	Sea	400 m × 400 m × 400 m	20 m	3 kHz	RF, Acoustic	Static	20 m	4
[127]	Surveillance	Ocean	Up to 3 km	90 m to 98 m	25.6 kHz	RF, Acoustic	Static, Mobile	No Info	7
[128]	Exploration	Sea	600 m × 900 m	Up to 80 m	3 kHz	Acoustic	Mobile	75 m	2
[129]	Ocean Sampling	Ocean	500 m × 500 m	10 m	2 kHz	Acoustic	Mobile	10 m	2
